# Redefining text-to-SQL metrics by incorporating semantic and structural similarity

**DOI:** 10.1038/s41598-025-04890-9

**Published:** 2025-07-01

**Authors:** Giovanni Pinna, Yuriy Perezhohin, Luca Manzoni, Mauro Castelli, Andrea De Lorenzo

**Affiliations:** 1 University of Trieste, 34127 Trieste, TS Italy; 2 NOVA Information Management School (NOVA IMS), Universidade NOVA de Lisboa, 1070-312 Lisboa, Portugal

**Keywords:** SQL metric, Evaluation metric, Text-to-SQL, Benchmark SQL, SQL similarity, Computer science, Computational science

## Abstract

The rapid advancements in text-to-SQL systems have driven the scientific community to create increasingly complex benchmarks for this task. However, evaluation metrics often rely on simplistic or binary approaches that fail to capture the similarities and differences between equivalent SQL queries. Current metrics overlook critical aspects such as partial correctness, structural differences, and semantic equivalence. To address these limitations, we propose a novel metric for SQL query comparison, designed to offer a more precise assessment of the similarity between SQL queries at both the semantic (string) and execution result (resultant table) levels. This new metric allows for a granular evaluation of SQL query similarity, supporting a more accurate assessment and ranking of text-to-SQL tools and models. The proposed approach could have a meaningful impact on text-to-SQL research and development. It might improve evaluation by distinguishing between models that handle simple queries and those capable of tackling more complex ones. The metric could also help to identify where the differences between two queries lie. Additionally, it may support the development of more accurate language models by offering precise training signals to help the model recognize query similarities. The experimental results highlight the metric’s effectiveness over existing evaluation methodologies, allowing us to identify the current best text-to-SQL models through distribution analysis. In some cases, the metric allows the detection of missing aggregation operators or variations in query ordering operators.

## Introduction

Natural Language Processing (NLP) has seen significant advances in recent years, with remarkable progress in generating SQL queries starting from a textual description (a task called *text-to-SQL*). The rising demand to make database interactions accessible to non-technical users has primarily driven this growth. Non-technical users can now retrieve information from databases using natural language instead of structured query languages^1,2^. In business intelligence, accurate SQL generation is essential for data-driven decision-making by allowing users to query complex datasets without deep technical expertise^3^. Similarly, in data analysis, the ability to quickly and reliably extract insights from large datasets through natural language requests improves efficiency and accessibility^4^. In customer support, natural language interfaces facilitate the swift resolution of user issues by allowing support staff to query databases directly, improving response times and customer satisfaction. These applications highlight the need for precise and adaptable text-to-SQL models to handle more complex queries and adapt to diverse real-world scenarios. Consequently, developing nuanced evaluation metrics and robust benchmarks has become crucial. Metrics are essential to accurately assess system performance, guide further innovation, and ensure the reliability of text-to-SQL systems in different applications and domains.

The rapid advancement of text-to-SQL capabilities has led the research community to design increasingly challenging benchmarks such as BIRD^5^ or SPIDER 2.0^6^. Modern test suites include complex scenarios with multi-table queries, nested sub-queries, and aggregations. However, while benchmarks have grown in complexity, evaluation metrics have not evolved simultaneously, often remaining simplistic and binary^7,8^. In the past, current metrics were adequate to develop text-to-SQL tools, but their binary approach now fails to capture the range of semantically equivalent but syntactically varied queries that achieve the same intended result. Furthermore, the focus on the correctness of the query does not address a key challenge: generating the most efficient SQL among valid alternatives.

Most current evaluation methods still rely on a binary framework that classifies SQL queries as exact matches or complete mismatches. Although straightforward, this approach overlooks the subtle distinctions between semantically equivalent queries despite syntactic differences or partial correctness. Consequently, traditional metrics may penalize models that produce valid alternative query formulations, highlighting the need for more refined evaluation metrics.

In any scientific domain, metrics provide a critical foundation for comparative analysis, allowing researchers to make informed judgments about advances, challenges, and potential directions for further research. Effective metrics not only facilitate meaningful evaluations of tools and models but also highlight the unique strengths and limitations of different approaches, guiding the evolution of the field.

To address these limitations, we introduce a novel metric that evaluates two key dimensions: the semantic similarity of SQL query structures and the equivalence of their execution results. Semantic similarity is measured by embedding the two queries and calculating their similarity score, capturing how similar they are in terms of structure and meaning. For table comparison, a set of predefined rules assesses the similarity of the queries output, producing a score that reflects the similarity of resulting tables in terms of content and structure. The two similarity scores are then combined into a final relevance score, allowing for a more granular and comprehensive assessment of the similarity of the queries while capturing both partial correctness and valid alternative formulations.

The proposed metric is particularly valuable because it provides detailed feedback rather than a simple binary outcome. Instead of merely indicating whether a query is correct or incorrect, it reveals how close a generated query is to the correct solution in terms of semantic structure and execution results. This level of granularity allows researchers to assess SQL query similarity more precisely, offering a richer evaluation of model performance. For instance, the metric can detect cases where two queries yield almost identical results at the table level despite slight differences in their semantic structure or vice versa -situations that would typically be marked as mismatches by conventional metrics. The proposed evaluation framework not only distinguishes between competing text-to-SQL models but also encourages the development of advanced SQL embedding techniques to identify semantically equivalent queries accurately.

The lack of granularity in current metrics for evaluating SQL queries similarity, particularly in text-to-SQL tasks, has significant implications for practical applications. When metrics fail to capture subtle differences in SQL query formulation, the following problems may occur:

1. Overlooking critical errors: metrics that fail to distinguish between errors of different severity may treat a missing condition or structural defect equivalently to a minor syntax problem. This limits the ability of developers or systems to prioritize and fix higher-impact problems, such as queries that are invalid or return incorrect results. The inability to distinguish the severity of errors during the training of text-to-SQL models may prevent weighted training feedback that accounts for variations in query discrepancies and can affect the quality of model training.
2. Obstacle to fine-tuning: text-to-SQL systems often require specific tuning. Without granular metrics highlighting specific areas for improvement, such as error categorization (syntactic, logical, or semantic problems), it becomes difficult to understand where models present difficulties and how to adjust training data or improve model architectures effectively.
3. Challenges in evaluating contextual understanding: text-to-SQL tasks often require understanding subtle user intentions expressed in natural language. Granular metrics help to assess whether the system captures nuances such as implicit constraints, references to specific columns, or contextual relationships between tables. Without them, models may be overestimated or underestimated in their capabilities. A non-binary score helps to understand how close a model-generated output is to the desired one, and thus indirectly how well it captures the implicit intentions of the natural language request provided by the user.

These reasons highlight how insufficient granularity in evaluation metrics can obscure true model performance, preclude targeted improvements, and ultimately reduce the effectiveness of text-to-SQL systems in real-world applications. Developing metrics, such as the one we proposed in this paper, that account for syntactic, semantic, and table-level differences is crucial to advancing this field.

## Related work

Allowing non-technical users to interact effectively with databases has long been a challenge in the field of NLP^2,8^. Existing text-to-SQL models can be categorized into three main approaches: rule-based methods, fine-tuning methods, and In-Context Learning (ICL) methods.

The first models for the text-to-SQL^9,10^ task were primarily based on rules, relying on predefined templates and rules to generate SQL queries. Although these models demonstrated reasonable performance, they were challenging to adapt to specific domains as they required extensive and time-consuming customization of templates and rules. Consequently, research in this field gradually moved towards sequence-to-sequence (Seq2Seq) models, relying on architectures like Long Short-Term Memory (LSTMs)^11^ and Convolutional Neural Networks (CNNs)^12^ to address the limitations in adaptability and flexibility that constrained rule-based models. However, despite these advances, Seq2Seq models continued to face challenges due to the structure of complex databases, which imposed persistent limitations. To overcome these constraints, Wang et al.^13^ and Cao et al.^14^ used graph neural networks (GNNs) to represent database schemas as graphs, allowing a more structured handling of complex schema relationships. Representing database schema as graphs is effective because graphs intuitively model relationships, enable efficient traversal and analysis, support visualisation, and align with modern database needs for flexibility and optimization. Meanwhile, other researchers have achieved high performance in the text-to-SQL task by fine-tuning pre-trained models such as T5^15–17^, as well as newer models such as DAILSQL^7^ and CodeS^18^. Despite these relevant results, fine-tuning methods rely heavily on large amounts of high-quality labeled data; in the absence of such data, these models risk overfitting the training set, limiting their generalizability.

Currently, there is a large interest in the use of Large Language Models (LLMs) for a large set of tasks^19–25^. These models outperform many fine-tuned models in various NLP tasks. Moreover, due to their ICL capability^19^, LLMs can further improve output quality through prompt engineering^26–30^. ICL enhances the flexibility of LLMs by allowing them to infer task requirements and patterns directly from the prompts they receive, without the need for explicit fine-tuning. This capability is particularly valuable for zero-shot tasks, where the model must generate appropriate responses based solely on the provided context. Moreover, prompting has several advantages compared to traditional approaches that use fine-tuning. The main advantage of prompt engineering in LLMs is that they can perform prediction tasks without requiring large task-specific training data. Training models from scratch or fine-tuning them is a resource-intensive process, often requiring a large number of training samples and machine resources, which may not be available. The C3 model^31^ exemplifies the effectiveness of ICL, particularly in generating zero-shot SQL queries with high accuracy.

To achieve the impressive results of modern text-to-SQL models, the development of high-quality benchmarks to effectively evaluate and rank model performance was fundamental. Early benchmarks in this field were primarily single-domain datasets^9,32,33^, whereas more recent benchmarks, such as WikiSQL^34^ and Spider^35^, are cross-domain, thus offering a broader test for model generalizability. However, most cross-domain text-to-SQL datasets still focus on the database schema rather than data values^36–39^, which can limit their alignment with real-world applications. The Spider benchmark^35^, one of the most widely used benchmarks in text-to-SQL research, introduced two of the most important metrics to evaluate text-to-SQL tools: Exact Match (EM) and Execution Accuracy (EX). These two metrics are fundamental for evaluating text-to-SQL models. EM assesses whether the SQL string generated by the model matches the reference SQL string, returning a binary value to indicate an exact or not-exact match. On the other hand, EX focuses on the equivalence of the query execution results rather than the query syntax. Since multiple SQL queries can produce the same result, EX checks if the tables obtained by executing both the reference and model-generated queries have the same set of values, yielding a binary result.

While these benchmarks have been adopted widely, recent research has highlighted critical limitations in their ability to fully capture model performance, particularly in cross-domain text-to-SQL tasks^40^. Researchers have introduced three advanced benchmarks (BIRD^5^, UNITE^41^, and ScienceBenchmark^42^) to address these gaps and better assess the capabilities of modern models. The main reason for creating these new benchmarks is to bridge the gap between academic research and real-world applications by introducing large databases with many complex tables and queries that require outside knowledge and that optimize the efficiency of SQL execution. In particular, recent benchmarks emphasize that current LLMs are not yet suitable as database interfaces for real-world applications. BIRD^5^, ScienceBenchmark^42^, and UNITE^41^ benchmarks are now regarded as the new state-of-the-art in text-to-SQL evaluation.

The BIRD benchmark^5^ introduces a novel evaluation metric called the Valid Efficiency Score (VES), which evaluates the efficiency of SQL queries based on their execution time. Furthermore, BERTScore (${\text{BERT}}_{{{\text{score}}}}$)^43^ has been used to go beyond simple similarity measures and to evaluate the semantic similarity between queries. ${\text{BERT}}_{{{\text{score}}}}$ captures semantic intent by relying on embeddings that measure the similarity between pairs of text^44^. Another common approach for calculating semantic similarity is using cosine similarity between vectors generated by Bidirectional Encoder Representations from Transformers (BERT) like models, which are designed to produce embeddings that encapsulate the semantics of text. While BERT models excel at capturing underlying intent through language-based representations, they are not specifically tailored to SQL. As a result, they may fail to capture critical structural, semantic, and logical aspects unique to SQL queries.

Despite these critical issues, the aforementioned metrics are widely used to evaluate text-to-SQL models.

## Method

The proposed metric evaluates the similarity between queries by considering two key aspects: semantic similarity and result-based similarity. This dual focus is crucial since queries with different syntactic structures can produce identical execution results.

**Fig. 1:**
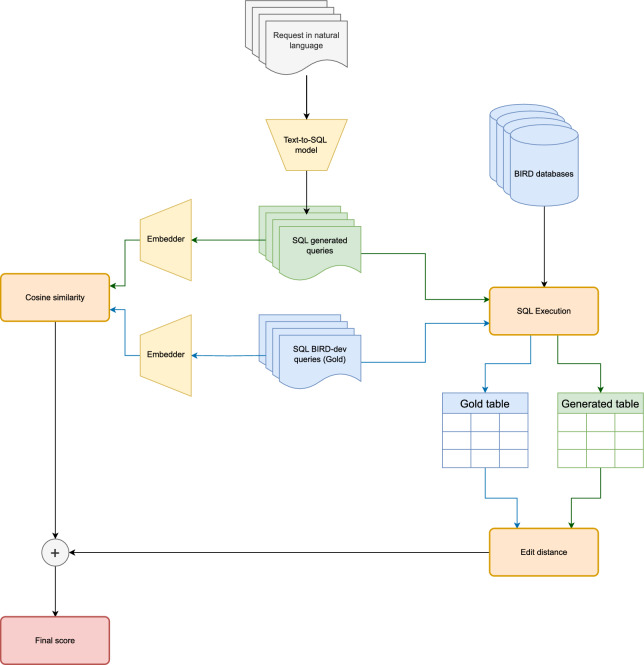
High-level pipeline of our approach for comparing SQL queries generated via a text-to-SQL method to the corresponding reference queries on the BIRD databases.

Figure 1 shows the considered pipeline. In particular, one can see that natural language requests, shown at the top center in gray, are passed to a text-to-SQL model to generate their respective SQL queries. The natural language prompts come from the dev branch of the BIRD benchmark. After obtaining the SQL queries from the text-to-SQL system and the corresponding reference queries, it is possible to compute the metric. Figure 1 shows three boxes, where each box schematizes the steps to calculate the components of the proposed metric. Box number one has all the components needed to compute the part of the metric concerned with semantic similarity. Box number two shows how to calculate the table-level similarity. Finally, box number three shows how to calculate the final value of the metric by taking a weighted sum of the values from boxes one and two. In this way, the final score, which we will call Query Affinity Score (QAS), will consider both the semantic similarity and the similarity between execution results.

### Semantic similarity ($S_C$)

The aim of analyzing semantic similarity, represented in box one in Fig. 1, is to determine whether different queries serve the same purpose and whether their structure is inherently similar. As discussed in Sect. 2, existing approaches, such as computing the cosine similarity between embeddings or using the ${\text{BERT}}_{{{\text{score}}}}$ method, return scores based on semantic similarity. However, these methods are not tailored for code, making them less effective in assessing the similarity of syntactically different but semantically equivalent queries.

To address this limitation, we use embedding models specifically designed for code, including SQL queries. These models, built on the same principles as BERT, are fine-tuned for programming languages. This specialization allows them to produce more consistent and reliable similarity scores than general-purpose models.

The embedding models work by mapping text, in this specific case, queries, into dense vectors in a vector space, ensuring that semantically similar queries are placed closer together in the vector space. This representation allows for a fine-grained comparison of the intent and underlying structure of the queries. Choosing a specifically designed embedding model for the code ensures that domain-specific nuances, such as the meaning of SQL keywords, use of subquery, aliases, and, more generally, the equivalence of different query formulations, are accurately captured and considered. Moreover, embedding models designed for code significantly improve the widely used EM metric. The latter checks if two queries are identical at the string level, overlooking that a query can be written in multiple syntactically different ways while accurately reflecting the same user intention. After computing embeddings using the selected models (see Sect. 4), the similarity between the resulting vectors is calculated using the cosine similarity.

Formally, this can be denoted as having an embedding $\mathcal {E}$ mapping a query to a vector in $\mathcal {R}^n$, where *n* is the dimensionality of the embedding. Given two queries $q_1$ and $q_2$, their semantic similarity is:

$$\begin{aligned} S_C (q_1, q_2) = \frac{\mathcal {E}(q_1) \cdot \mathcal {E}(q_2)^T}{\Vert \mathcal {E}(q_1) \Vert \; \Vert \mathcal {E}(q_2) \Vert } \end{aligned}$$

That is, as stated above, the cosine similarity between the embeddings of the queries.

### Table similarity ($S_T$)

The second key aspect of the proposed metric is the similarity between the result tables obtained by executing the queries. We can observe a graphical representation of this part in box two of Fig. 1. In the current benchmarks, this aspect is addressed using the EX metric, which returns a binary value: 1 if the two tables are identical and 0 otherwise. This simple approach lacks granularity and fails to quantify the degree of difference between the result tables. To address this limitation, we propose a more refined method, which evaluates table similarity first by checking if the generated SQL query and the reference SQL query are identical strings. If the strings are identical, by definition, the tables produced by these queries will also be identical, making their similarity score equal to 1. If the strings differ, we execute both SQL queries on the database used by the reference query. If the generated query cannot be executed, we set the similarity to zero by default. Otherwise, we compare their results as follows.

Let $T_{gen}$ and $T_{ref}$ represent the result tables produced by the generated query and the reference query, respectively. For each pair of columns $(c_i, b_j)$, where $c_i \in T_{gen}$ and $b_j \in T_{ref}$, we calculate the edit distance required to transform $c_i$ into $b_j$. This distance is defined as the minimum number of replace, delete, and insert operations necessary to align the two columns. The overall table similarity score ($S_T$) between $T_{gen}$ and $T_{ref}$ is then calculated as the normalized aggregation of the edit distances across all column combinations. Specifically, for *n* columns in $T_{gen}$ and *m* columns in $T_{ref}$, we define:

$$\begin{aligned} S_{\text {T}} = 1 - \frac{1}{\max (n, m)} \sum _{i=1}^n \frac{\min _{j=1}^m d(c_i, b_j)}{\max (len(c_1), len(b_1))}, \quad \forall i = 1,2,...,n \quad \text {and}\quad \forall j = 1,2,...,m \end{aligned}$$

That is, we normalize the minimum edit distance for each column pair by dividing it by the maximum column length—i.e., $\max (len(c_1), len(b_1))$—of the tables, ensuring scale invariance. By definition, each column in the generated table contains an equal number of rows, and the same applies to the reference table. To determine the maximum number of rows between the two tables, we take the larger value between the number of rows in column zero of the generated table $len(c_1)$ and the number of rows in column zero of the reference table $len(b_1)$. These normalized distances are aggregated across all column pairs, and the table score is calculated as one minus the normalized distance derived by the maximum number of columns between the two tables. The result is the similarity measure $S_T$ and ranges in the interval [0, 1], where 1 represents identical tables and 0 represents complete dissimilarity. This algorithm is robust to variations in column alignment and data length, allowing for meaningful comparisons across diverse table sizes.

### Query affinity score (QAS)

The calculation of the final metric, called Query Affinity Score (QAS), is depicted in box three of Fig. 1. This score reflects the similarity between SQL queries by integrating both their semantics and execution outcomes. QAS is computed as a weighted average of the $S_C$ and $S_T$ components. Formally, the QAS score is defined as:

1 $$\begin{aligned} \text {QAS} = (1-w) \cdot S_{C} + w \cdot S_{T} \end{aligned}$$

where *w* ($0 \le w \le 1$) is a weighting factor that balances the contributions of the two components. The similarity score QAS ranges between 0 and 1, as it is calculated as the weighted sum of two measures, $S_T$ and $S_C$, which also provide scores within the [0, 1] interval.

## Experiments

To evaluate the proposed metric, we performed experiments using the branch-dev subset of the BIRD benchmark^5^. We selected BIRD due to its optimal balance between realistic size, complexity, and representativeness, which ensures the robustness of our experiments. In fact, BIRD is one of the benchmarks for the text-to-SQL task that most closely approximates real-world scenarios.

The evaluation framework considers outputs from 11 diverse text-to-SQL models:

- C3 SQL ^31^,
- DAILSQL ^7^,
- DAILSQL SC ^7^,
- RESDSQL 3B EK ^17^,
- RESDSQL Base EK ^17^,
- RESDSQL Large EK ^17^,
- SFT CodeS 1B EK ^18^,
- SFT CodeS 3B EK ^18^,
- SFT CodeS 7B EK ^18^,
- SFT CodeS 15B EK ^18^,
- SuperSQL ^45^.

These models represent a range of architectural paradigms, such as fine-tuned models and ICL implementations. The outputs of these models were taken from publicly available results in GitHub repositories (https://github.com/giovannipinna96/NL2SQL360/tree/master/data/predict/bird_dev).

Baseline performance was assessed using the EX and EM metrics, both implemented via the official BIRD repository (https://github.com/AlibabaResearch/DAMO-ConvAI/tree/main/bird). For semantic analysis, we tested two specialized code embedding models: UAE-Code-Large-V1 (https://huggingface.co/WhereIsAI/UAE-Code-Large-V1)^46^ and all-MiniLM-RAGSQL-code (https://huggingface.co/sergeyvi4ev/all-MiniLM-RAGSQL-code). These models were fundamental for computing semantic and syntactic aspects of SQL queries accurately. The UAE-Code-Large-V1 model was selected because, with its innovative approach that reduces the negative impact of the saturation zone of the cosine function, it represents the new state-of-the-art for embeddings. Meanwhile, the all-MiniLM-RAGSQL-code model was included due to its popularity and proven effectiveness in generating embeddings. These models are robust tools for interpreting complex queries by leveraging their ability to map words and phrases into dense vector representations, thus allowing semantic understanding. Furthermore, they employ positional encoding and attention mechanisms to achieve syntactic awareness, learning the structure and relationships within sentences. By combining these capabilities, the models effectively interpret and understand natural language queries.

Since there is no *a priori* relative importance to be assigned to semantic and table similarity—i.e., the value of the parameter *w* in Eq. 1—we performed an initial testing for *w* ranging from 0 to 1 with a step of 0.25. Notice that the QAS scores obtained for different values of *w* are not directly comparable—i.e., they are different metrics. However, we compared the induced ranking of the queries and distribution of values to both understand the influence of *w* and select a reasonable value of it for the remaining experiments.

After a preliminary evaluation, we selected the UAE-Code-Large-V1 model for its superior performance in interpreting SQL queries. The model excelled in capturing the nuances of query structure and semantics. Additionally, it spaced query vectors more effectively in the vector space, resulting in a more distributed range of similarity scores between 0 and 1.

We conducted the experiments on hardware consisting of an NVIDIA A100 GPU (limited to 20 GB of VRAM), 8 CPU cores, and 128 GB of RAM. Computational efficiency was a critical consideration, as the time required for metric calculations may significantly impact the practical applicability of the proposed approach. Hence, the analyses include a time evaluation across the BIRD-dev benchmark, a comparative assessment of conventional metrics, and a detailed examination of the proposed metric over specifically designed test cases to evaluate its capabilities on challenging queries.

Given these settings, we conducted the following series of evaluations to assess the behavior and effectiveness of the proposed QAS metric:

1. first, we perform a study of the effect of the parameter *w*. We analyzed how different values of *W* affected the resulting rankings and similarity scores. This allowed us to select an appropriate balance between semantic similarity and table similarity for subsequent evaluations;
2. then we investigated the distributions of the semantic similarity, table similarity, and the combined QAS across the outputs of all considered models;
3. we compared the proposed metric with the Exact Match (EM) and Execution Accuracy (EX) metrics to highlight differences in behavior, particularly in handling partial correctness and variations in query structure;
4. we measured the time required to compute the metric for queries of varying sizes, in order to assess its computational feasibility;
5. finally, we conducted targeted experiments on specially constructed queries to test the sensitivity of the metric to specific modifications, such as changes in the number of rows and columns or alterations in ordering clauses.

## Results

This section presents the analysis of the results obtained for the experiments outlined in Sect. 4. In particular, in Sect. 5.1 we study the influence of the parameter *w* in the computation of the QAS. In Sect. 5.2 we explore the distribution of $S_T$ score, while the distribution of QAS is studied in Sect. 5.3. An analysis of the time needed for the computation of the $S_T$ scores is presented in Sect. 5.4. Finally, an in-depth analysis of particular cases is explored in 5.5

### Selection of the parameter *w*

To systematically characterize the sensitivity of the weighting parameter *w*, we carried out a comprehensive comparative analysis across multiple values of *w*. The objective was to identify a configuration that achieves a balanced integration of semantic and tabular similarities. To this end, rankings generated under different *w* values were compared using the Kendall distance^47^, a widely adopted metric for quantifying the degree of dissimilarity between permutations. Higher Kendall distances reflect greater discrepancy between rankings, while lower values indicate stronger concordance. The following analyses elucidate the patterns of ranking variation observed as a function of *w*, ultimately supporting a principled selection of the most appropriate weighting scheme for subsequent experiments.

**Fig. 2:**
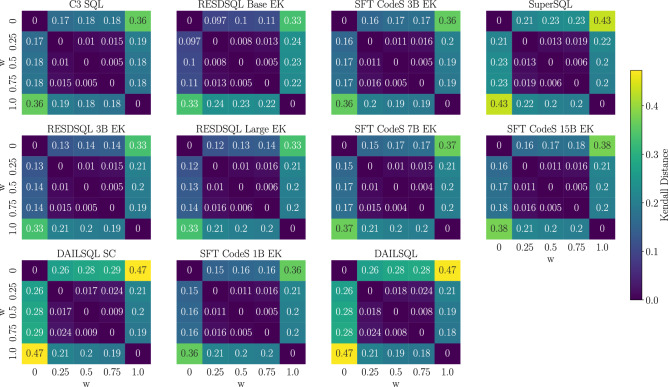
Kendall distances between different models as a function of the weight *w*.

**Fig. 3:**
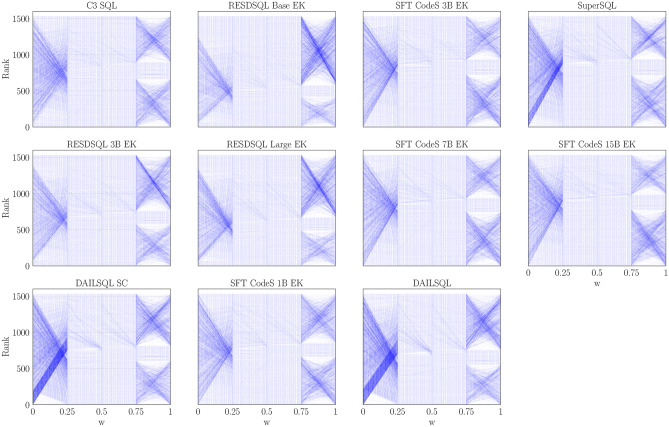
Query ranking shifts across different values of the weighting parameter w. Lines connect the same query across w values, with darker blue indicating larger rank changes.

Figure 2 presents a heatmap summarizing the Kendall distance across different models, computed for various weight parameter values *w*. Each cell represents the distance between the rankings obtained with two distinct values of *w*. Consistently across all models, the distances are larger when comparing rankings generated with $w = 0$ and $w = 1$, as these two settings emphasize exclusively tabular similarity and semantic similarity, respectively. Intermediate values of *w* (i.e., 0.25, 0.50, and 0.75) yield much smaller distances when compared among themselves, indicating smoother and more gradual changes in the resulting rankings. These observations suggest that when *w* lies in the intermediate range, the ranking results are more stable, reflecting a balanced contribution from both semantic content and tabular structure. Based on this analysis, $w = 0.5$ was selected in our experiments to equally weight the two components without privileging one aspect over the other.

Figure 3 complements this analysis by showing the rank evolution of queries as a function of the weight parameter *w*. Each line tracks the ranking of a specific query across different values of *w*, with the color intensity reflecting the magnitude of ranking changes between consecutive settings–darker lines indicating more substantial shifts. The plots reveal a common trend across all models: significant ranking variations occur primarily in the transitions from $w = 0$ to $w = 0.25$ and from $w = 0.75$ to $w = 1$, corresponding to the extremes where a single similarity component dominates. Conversely, rankings appear relatively stable across the intermediate values ($w = 0.25$, 0.5, and 0.75), further supporting the view that these configurations promote a smoother and more balanced adjustment. These findings reinforce the choice of $w = 0.5$ to achieve an equilibrium between semantic and tabular information.

### Distribution of $S_T$ score

**Fig. 4:**
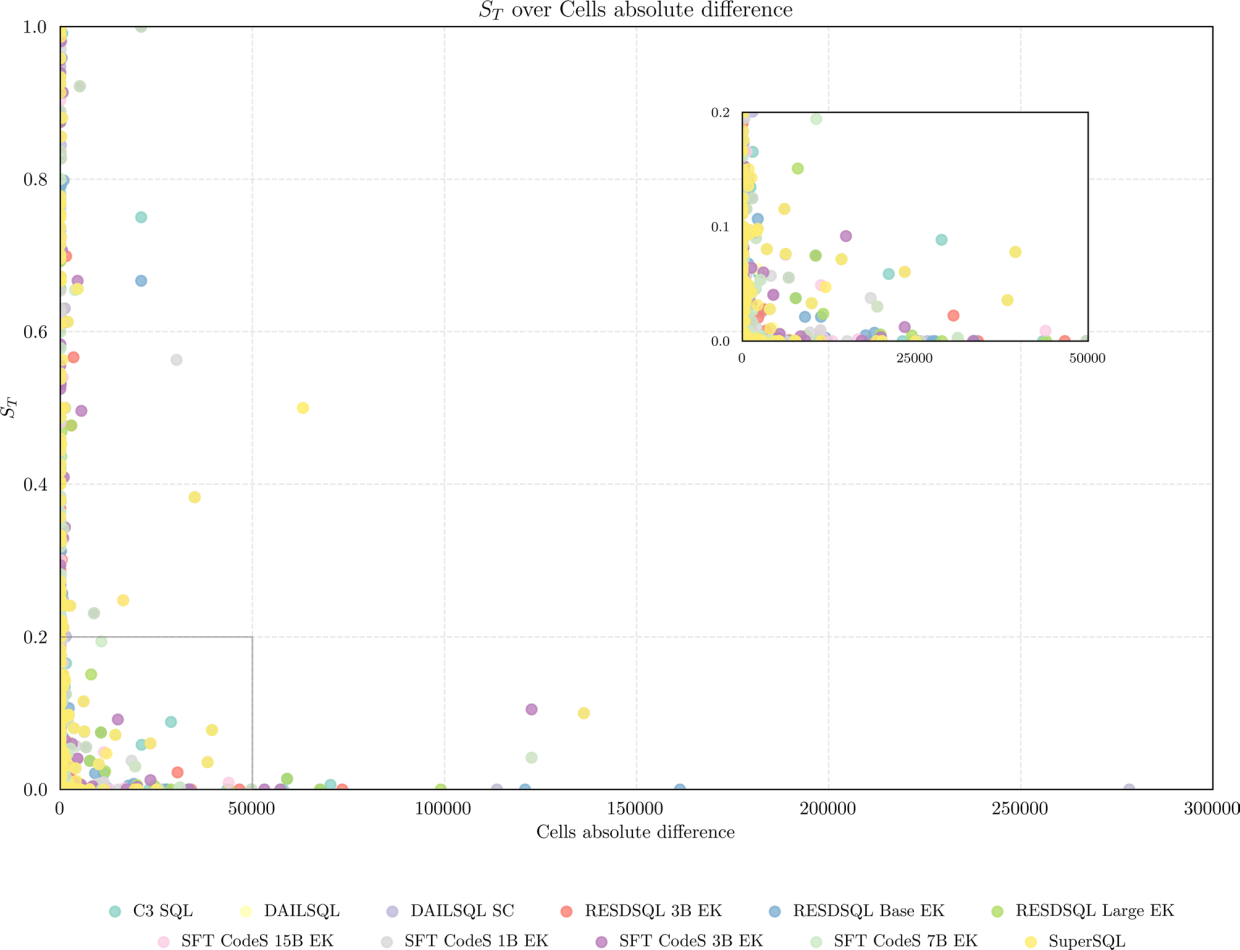
Correlation between $S_T$ and the difference in the number of cells between the two tables.

To emphasize the significance of the proposed metric, Fig. 4 illustrates the distribution of the $S_T$ score against the absolute difference in the number of cells between the reference and generated tables. These results demonstrate that merely comparing the shapes of the tables provides no meaningful insight into their actual similarity. Figure 4 demonstrates that tables with minimal or no difference in the number of cells can exhibit $S_T$ scores spanning the entire range [0, 1]. This variability highlights the absence of a consistent relationship between $S_T$ and the difference in the number of cells between tables. Moreover, this pattern is consistent across all models, as indicated by the different colors representing each model in Fig. 4. Additionally, it is worth noting that when the difference in the number of cells exceeds 50000, the similarity score is consistently zero or near zero. However, such extreme differences are rare, as most tables have comparable sizes but exhibit a wide range of similarity values. This highlights the importance of developing a new metric that accounts for additional aspects of SQL queries. Simpler methods, such as comparing the difference in the number of cells, fail to provide sufficient information about the similarity of SQL queries.

The $S_T$ score offers significant advantages over traditional binary metrics, such as EX, and overly simplistic methods. Unlike binary metrics, the proposed approach generates well-distributed similarity scores across the [0, 1] range, allowing for a more nuanced and comprehensive evaluation.

**Fig. 5:**
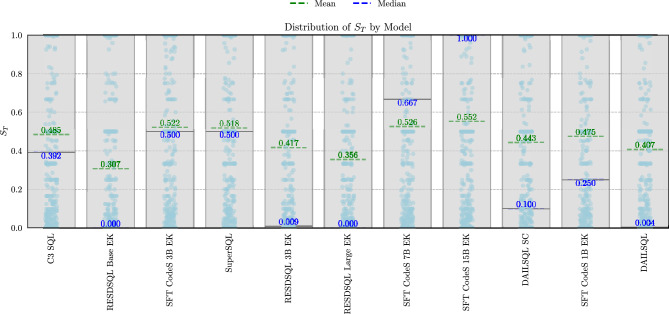
Boxplot showing the distribution of $S_T$ scores for each of the 11 models.

Figure 5 presents 11 boxplots, each representing the distribution of $S_T$ scores obtained from queries generated by a specific text-to-SQL model. In the boxplots, the light-blue dots represent the scores obtained for a specific pair of reference and generated queries by the corresponding model. The green and blue dashed lines indicate the mean and median values for the respective models. Notably, in many models, the median is not at the extreme values (0 or 1) but is positioned closer to the middle of the distribution. This highlights how current metrics, such as EX, often classify many results as incorrect, even when they are partially correct, or vice versa. It suggests that erroneous results may still contain some information relevant to the natural language request. This increased granularity allows more nuanced and accurate comparisons between models. Using the proposed metric for a single query, it becomes evident that a similarity value within the set of real numbers is far more informative than a binary value. The metric also facilitates more precise comparisons between text-to-SQL models by incorporating both the mean and median. With non-binary similarity scores, the mean and median can be calculated more accurately, offering a more comprehensive representation of the models’ true capabilities. For example, although the C3 SQL and SFT CodeS 1B EK models in Fig. 5 exhibit similar average performance, SFT CodeS 1B EK has a significantly lower median performance. This makes C3 SQL the better model of the two. Another comparison reveals that while the SFT CodeS 7B EK and SFT CodeS 15B EK models exhibit comparable mean performance, SFT CodeS 15B EK demonstrates a higher median accuracy, making it the better-performing model overall. Supporting this claim is the design of the models in this particular case. Specifically, SFT CodeS 7B EK has fewer parameters than SFT CodeS 15B EK, and since both models belong to the same family, it is expected that the version with more parameters would achieve better results.

**Fig. 6:**
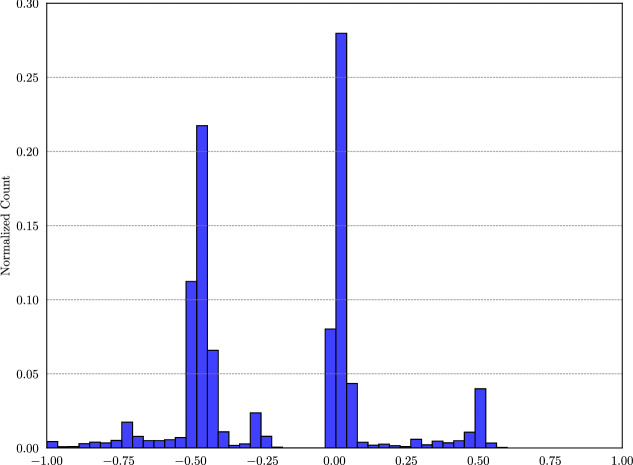
Comparison of the proposed approach with EX. The x-axis represents the magnitude of the difference between EX and the proposed metric.

The comparative analysis between EX and the proposed metric reveals several significant patterns. The graph in Fig. 6 illustrates the distribution of the differences between EX and the proposed metric, calculated as (EX- QAS). On the x-axis, the graphs represent the difference between EX and QAS, while the y-axis indicates the number of reference and generated query pairs (across all models) corresponding to each level of difference. Positive values on the x-axis show that the majority of differences are concentrated around 0, with a secondary peak near 0.5. This portion of the distribution highlights the binary nature of EX and reveals instances where it mistakenly assigns perfect scores. Such cases typically occur when the generated tables include duplicate values that correspond to a single correct entry. The negative skew in the distribution, centered around -0.5, further illustrates EX ’s tendency to assign zero scores in cases where the proposed metric recognizes partial correctness. A tail extending from -0.5 to -1 is also evident, highlighting EX ’s tendency to assign zero scores to partially correct results.

### Distribution of query affinity score (QAS)

**Fig. 7:**
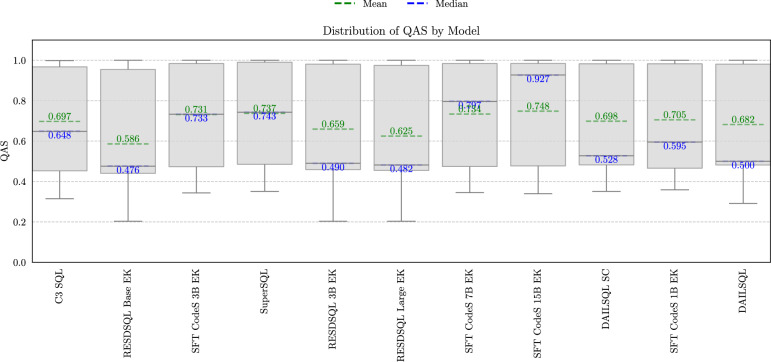
QAS distribution across all the models.

QAS is computed as the weighted sum of $S_T$ and $S_C$. Figure 7 shows the QAS distributions for each model, with the green and blue lines representing the mean and median values, respectively. The graph shows a significant increase in mean and median values compared to the distribution based exclusively on $S_T$, as reported in Fig. 5. This trend suggests that the models generate queries that are semantically well-aligned with those in the BIRD benchmark. Adding the semantic similarity component does not lead to significant changes in the ranking of the models compared to Fig. 5. Moreover, all distributions consistently include values above 0.2, indicating that while many queries produce significantly different tables, their semantics remain closely aligned with the reference queries.

An interesting insight from the graph is the variability of scores for each model, as depicted in the box plots. Although the RESDSQL 3B EK, RESDSQL Large EK, and DAILSQL models exhibit similar means and medians, indicating comparable overall performance, a closer analysis of their variability reveals differences in robustness. The DAILSQL model demonstrates a narrower range of variability compared to RESDSQL 3B EK and RESDSQL Large EK. In particular, the DAILSQL model maintains a minimum score of 0.3, whereas the other two models reach a lower minimum score of 0.2. This reduced variability and higher minimum performance highlight the greater consistency and robustness of the DAILSQL model, making it the most reliable option among the three models considered.

### Time analysis

**Fig. 8:**
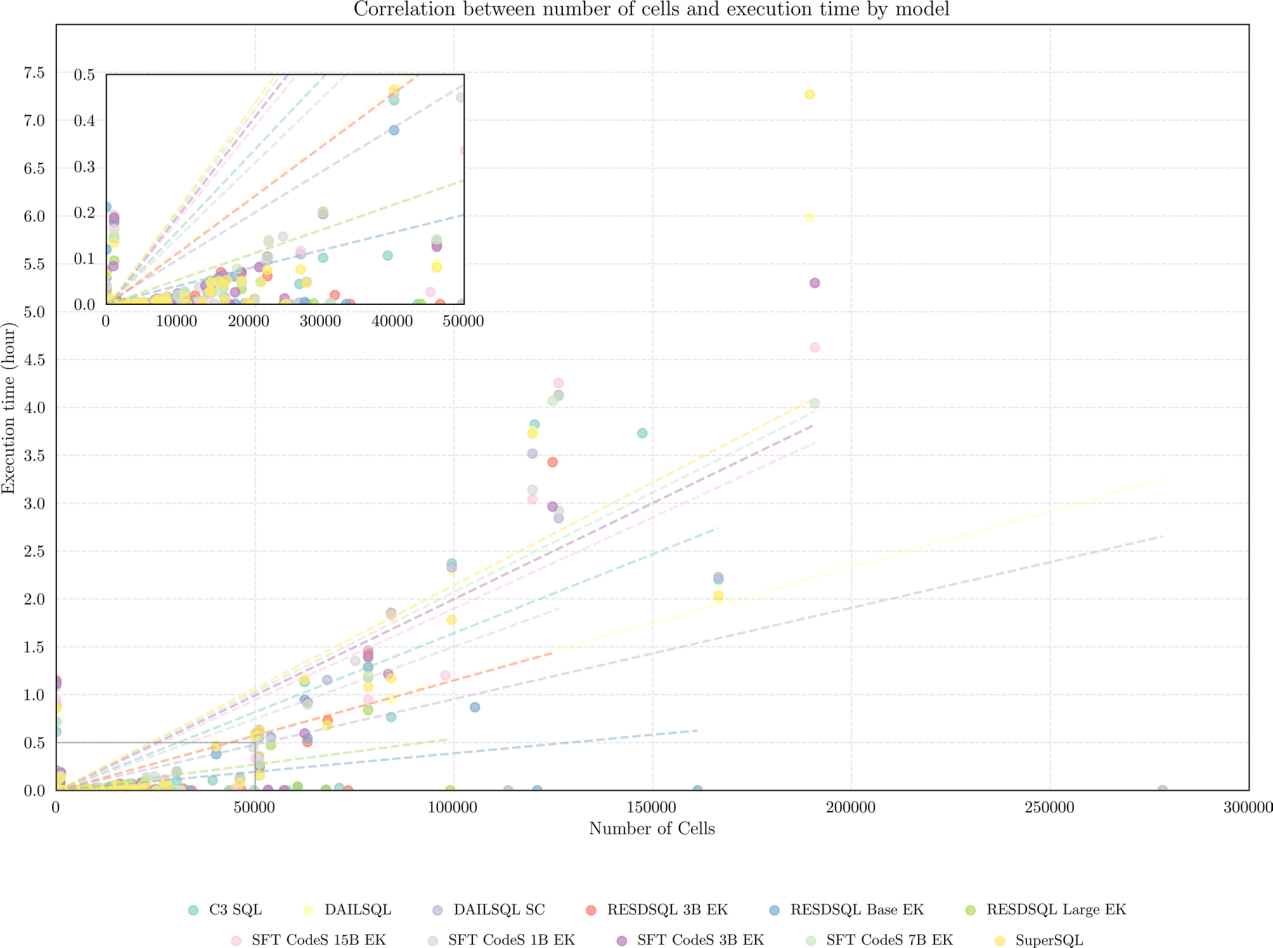
Graph showing the computation times for calculating the $S_T$ metric. The y-axis represents time in minutes, where each increment of 0.5 corresponds to 30 minutes.

Figure 8 illustrates the computation time required to calculate $S_T$ scores as the total number of cells in the tables increases. To provide a clear visualization and account for the sizes of both tables, the x-axis represents the sum of the number of cells in the tables. The y-axis indicates the computation time, where each increment of 0.5 corresponds to 30 minutes. Different models are distinguished by color, and dashed lines depict the trends in computation time as the number of cells grows.

Notably, for the vast majority of queries, the table similarity calculation takes less than a minute, primarily because the number of cells is typically fewer than 1000. However, we identified rare instances where processing times can extend up to 4 hours, with a single outlier requiring 7.3 hours. A key observation from the graph is that, as expected, the computation time increases linearly with the growth in the number of cells.

This is due to the design of the $S_T$ metric, which compares rows and columns. As the number of rows and columns increases, the number of comparisons grows proportionally, leading to longer computation times. The relationship between execution time and the number of cells generally exhibits a linear trend across most models, with notable deviations observed in the RESDSQL Base EK and RESDSQL Large EK models. In Fig. 8, the dots representing execution times close to zero, despite a large number of cells, correspond to cases where the SQL query strings were identical. In these instances, the table similarity is 1 by definition, eliminating the need for any comparisons.

### Analysis on edge cases and particular SQL scenarios

To assess the ability of the metric to differentiate between various scenarios, we conducted an evaluation using variants of a reference query on the *california_school* database provided by the BIRD benchmark. Then, we applied the metric to measure the difference between the reference query and each of its variants. In our experiments, we chose Query 1 because it includes a diverse set of operators. This query returns a single-row, single-column table due to the presence of a limit clause. The result identifies the mailing address with the highest number of K-12 students, determined by a descending sort. To measure all the components of the proposed metric, Query 1 was rewritten by replacing the JOIN operation with a subquery: this way, the result remains identical, but the query text is slightly different. Moreover, this allowed us to have greater control over the query to create its variants (using a subquery makes it possible to modify both the number of rows and the sorting of the results). Ultimately, we created 6 groups, each containing 4 variants of Query 1, resulting in 24 queries. The variations applied to each group are as follows:

**Column Number**: This group evaluates the metric as the number of columns increases. Additional columns are incrementally added to the variants of Query 1, selected iteratively from the available columns in the *california_school* database.
**Row Number**: This group examines the behavior of the metric with increasing rows. Rows are added incrementally, from 1 to 4, by adjusting the argument of the LIMIT operator in the variants of Query 1.
**Columns with Additional Rows**: This group investigates how the metric responds when the number of columns increases from 1 to 4 while keeping the number of rows fixed at 4.
**Rows with Additional Columns**: This group evaluates the metric as the number of rows changes while keeping the number of columns fixed at 4. Variants are derived from a subquery version of Query 1, where the row count is incrementally increased by adjusting the LIMIT operator.
**Rows and Columns Combined**: In this group, both rows and columns are incrementally increased together, starting from one row and one column and progressing to four rows and four columns.
**Rows with Different Ordering**: This group explores how ordering affects the metric. Variants are created by changing the ORDER BY clause to ASC and incrementally increasing the number of rows from 1 to 4.

**Fig. 9:**
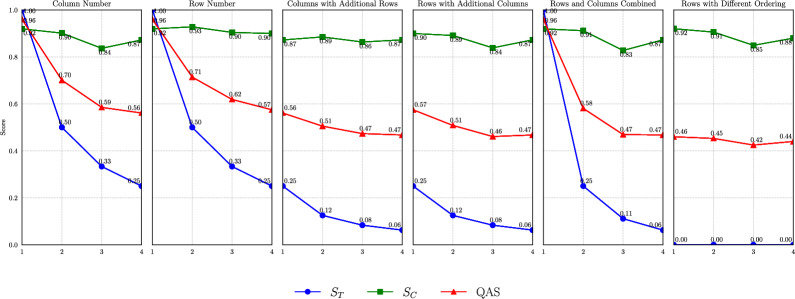
Graphs illustrating the trend of the QAS and its partial component scores, $S_T$ and $S_C$. The x-axis represents the *i*-th variation of Query 1 within each specific group.

Figure 9 illustrates the trends of QAS, $S_C$, and $S_T$ for each group and every query variation within the groups. The x-axis represents the query number within a specific group, while the y-axis indicates the similarity score. These experiments reveal that semantic similarity and table execution results, on their own, are not sufficient as evaluation criteria. For instance, in all groups except **Row Number**, the $S_C$ metric yields an unexpectedly higher score for the fourth SQL query variation compared to the third, despite the fourth variation deviating more significantly from the Query 1. This is evident from the rising trend of the green line ($S_C$) in Fig. 9 between the third and fourth positions of the specified groups. In contrast, the blue line ($S_T$) either continues to decrease or remains at zero. Since these queries were specifically constructed, we know that groups one through five (inclusive) contain queries that progressively deviate further from Query 1. Consequently, the fourth query variation is more diverse from Query 1 than the third. However, this difference is not effectively captured by the pipeline component responsible for calculating $S_C$. Conversely, the $S_T$ score in group **Rows with Different Ordering** consistently returns zero, even for meaningful structural variations in the query, such as changing the order (ASC to DESC). This highlights the need for a more nuanced scoring approach. This is why the proposed metric combines these two similarities in a weighted manner to compute the QAS. Further details about the SQL queries in groups **Column Number**, **Row Number**, **Columns with Additional Rows**, **Rows with Additional Columns**, **Rows and Columns Combined**, and **Rows with Different Ordering** are provided as supplementary material.

To further validate the robustness of the metric, we conducted an in-depth analysis of a set of queries specifically tailored to represent edge cases. This setup allowed a controlled evaluation of how the proposed metric responds to specific variations.

Query 2 is designed to evaluate the metric’s ability to capture semantic representation.

**Query 1:**
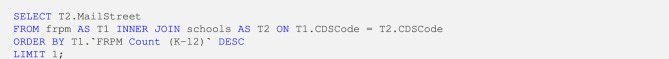
Reference query for edge cases analysis.

**Query 2:**
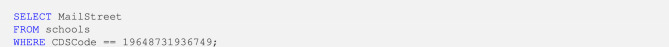
Query that produces the same result as the reference query but obtained through specific access to the record usingthe primary key.

This case demonstrates how identical result tables ($S_T$ = 1) can be produced by semantically distinct queries, as reflected by a low $S_C$ score (0.39). The generated query achieves the same result by leveraging primary key selection, highlighting the importance of considering both semantic and execution similarities in QAS computation for a comprehensive evaluation.

Query 3 illustrates the opposite scenario.

**Query 3:**
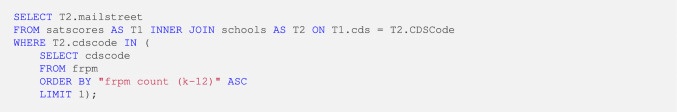
Query that returns the same table as the reference query but with opposite row order.

In this case, a high $S_C$ (0.97) coexists with divergent execution results caused by a reversed order (DESC replaced with ASC), resulting in a $S_T$ score of 0 despite the high $S_C$.

Query 4 demonstrates near-perfect alignment.

**Query 4:**
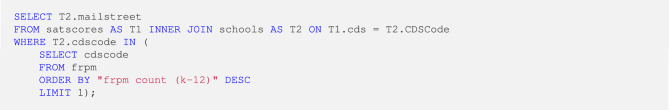
Query that exhibits a syntactic variation from the reference query while producing the same execution result.

In this case, $S_T$ equals 1 and $S_C$ achieves a high score of 0.97, highlighting the advantage of using code-specific embedding models over general-purpose alternatives.

Query 5 represents a typical partial match scenario.

**Query 5:**
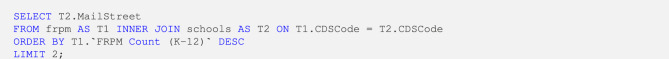
A query that partially matches the reference query’s result.

This example illustrates how the proposed metric handles partial matches, with a $S_T$ score of 0.5 reflecting the doubled row count in the generated query 5 while maintaining a high $S_C$ (0.98) due to minimal query modifications.

### Error identification in SQL queries

Another significant advantage of the metric is its ability to provide insights about possible key differences between two SQL queries.

**Query 6:**
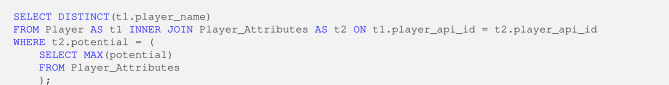
Reference query with DISTINCT aggregation operator

**Query 7:**
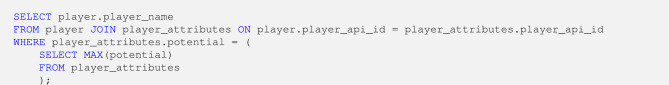
Generated query without aggregation operator.

We evaluated the proposed metric on Query 6 and the generated Query 7. The results show a $S_C$ of 0.95, a $S_T$ score of 0.16, and a corresponding QAS value of 0.55. Analyzing $S_T$ and $S_C$ separately, we observe that $S_T$ is significantly lower than $S_C$, indicating that while the two queries are semantically similar, their execution results differ considerably. This discrepancy highlights the substantial impact that seemingly minor differences in query phrasing can have on execution results. For example, such variations may arise from opposing sorting orders, different filtering conditions, or the absence of an aggregation operator, as seen in this case. A similar observation applies to the Query 10 and the generated Query 11. Here, the $S_T$ score is again notably lower than $S_C$, which can be attributed to the opposite sorting orders in the two queries.

However, interpreting query errors based only on the scores provided by the metric can be challenging. For instance, in the case of Query 8 and the generated Query 9, the scores exhibit the same pattern discussed earlier: $S_T$ is significantly lower than $S_C$, but for a different reason. Here, the error is not related to sorting keywords or aggregation operators. Instead, the key difference lies in how the zip code column is accessed. Query 8 uses a JOIN, which is absent in the generated query. Although both queries include a SELECT statement for the zip code, the results differ substantially due to the missing JOIN.

While the metric may not always provide precise explanations or pinpoint errors with absolute certainty, it remains a valuable tool for identifying potential issues. In contrast, traditional metrics such as EX and EM fail to offer meaningful insights. Across all the aforementioned cases, these metrics consistently return 0, providing no means to detect or analyze errors or any indication of their severity.

**Query 8:**
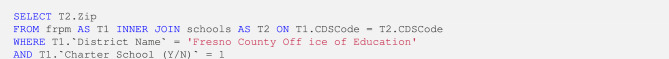
Reference query with JOIN operator between tables

**Query 9:**
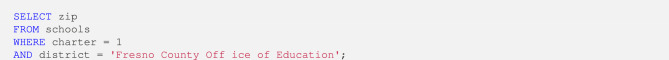
Generated query involving a single table.

**Query 10:**
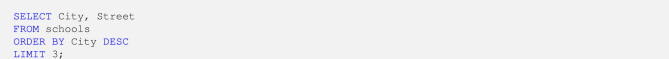
Reference query with descending sorting on City column.

**Query 11:**
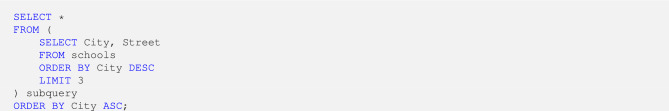
Generated query that returns the same table as the reference query but with rows sorted in ascending order.

## Discussion

Several important considerations can be drawn based on the results presented in Sect. 5. First, moving from a binary scoring system to a granular similarity measure allows for a more effective evaluation of the generated queries. This result is particularly relevant as it allows for more precise comparisons between text-to-SQL models. This analysis is facilitated when compared to existing metrics, which are limited to binary outcomes, significantly restricting their precision.

One potential advantage of using granular metrics is their ability to provide more precise feedback to the model during training. This feedback can improve the model’s performance by the end of the training phase. The proposed metric is essential for comparing models and identifying the most suitable one for real-world applications. For instance, it can help select the best model for generating SQL queries within a specific domain.

Another advantage of the proposed approach is that the computation of the QAS considers both the semantics of SQL queries and their execution results. As demonstrated in Fig. 9, relying solely on the two separate scores provides only a partial view of queries similarity. This limitation is evident, as different queries can produce the same result, whereas nearly identical queries may return opposite or significantly different outcomes, as in the case of reverse sorting. However, by accessing the individual similarity scores, it becomes possible to determine whether the query’s greatest similarity lies in its semantics or its execution result. Furthermore, this approach makes it possible to assess the error magnitude and the identification of its most probable source, as discussed in Sect. 5.6.

The final advantage of the proposed metric lies in its composition. Since QAS is a linear combination of the semantic similarity score and table similarity score, it allows for adjusting the importance and contribution of these two components. In our experiments, we assigned equal weights of 0.5 to $S_T$ and $S_C$, giving them equal significance in determining QAS.

Increasing the weight of $S_T$ can be advantageous when prioritizing execution results over semantic similarity, particularly in business scenarios where the outcome of the query is more critical than the query’s structure. Conversely, placing more emphasis on semantic similarity can be useful for tasks such as clustering queries or selecting queries for training models with contrastive losses, such as contrastive loss or triplet loss.

Despite these advantages, the proposed approach has some limitations, particularly regarding the time and computational resources required to compute the metric. In particular, as explained in Sect. 5.4, calculating the similarity between tables with a large number of rows and columns can be time and memory-demanding. During the experiments, we encountered 49 instances across all models where a similarity score could not be computed due to this limitation.

## Conclusion

This paper proposes a novel metric to evaluate queries from semantic and execution result perspectives. By integrating these two dimensions, the metric addresses the limitations of existing approaches that consider these aspects separately and thoroughly.

One of the significant enhancements introduced by the proposed metric is its ability to provide granular similarity scores. Unlike existing metrics, which return only binary outcomes (0 or 1) to indicate whether two queries are identical or completely different, the proposed metric evaluates the semantic similarity between two queries and their execution results. This capability helps identify whether discrepancies arise from differences in the query structure or the execution results. Furthermore, its design allows assessing how much relevant information is captured in the table produced by the generated query.

Despite these advancements, the current metric has limitations, particularly concerning execution time and memory consumption. Addressing these challenges will be the focus of future work. Specifically, we plan to develop a more relaxed version of the metric to improve efficiency and reduce resource demands. Additionally, we aim to enhance and parallelize the computation of table distances for better performance.

## Supplementary Information

Supplementary Information.

## Data Availability

All data used in this work are publicly available. The BIRD dataset used for evaluation is available at https://github.com/AlibabaResearch/DAMO-ConvAI/tree/main/bird. Additionally, the SQL queries predicted by the various text-to-SQL models analyzed in this paper are accessible at https://github.com/giovannipinna96/NL2SQL360/tree/master/data/predict/bird_dev.

## Declarations

### Code Availability

The code used in this study is publicly available at https://github.com/giovannipinna96/sql_metric under the MIT license. The repository’s README file provides detailed instructions for installing, using, and reproducing the results. For any inquiry regarding the code, please contact GIOVANNI.PINNA@phd.units.it.

## References

[1] Affolter, K., Stockinger, K. & Bernstein, A. A comparative survey of recent natural language interfaces for databases. *VLDB J.* **28**, 793–819 (2019).

[2] Őzcan, F., Quamar, A., Sen, J., Lei, C. & Efthymiou, V. State of the art and open challenges in natural language interfaces to data. In *proceedings of the 2020 ACM SIGMOD international conference on management of data*, 2629–2636 (2020).

[3] Zhu, J. et al. Talk to your data: Enhancing business intelligence and inventory management with llm-driven semantic parsing and text-to-sql for database querying. In *2023 4th international conference on data analytics for business and industry (ICDABI)*, 321–325 (IEEE, 2023).

[4] Guo, A., Zhao, X. & Ma, W. Er-sql: Learning enhanced representation for text-to-sql using table contents. *Neurocomputing* **465**, 359–370 (2021).

[5] Li, J. et al. Can llm already serve as a database interface? a big bench for large-scale database grounded text-to-sqls. *Adv. Neural Inf. Process. Syst.* **36**, 10245 (2024).

[6] Lei, F. et al. Spider 2.0: Evaluating language models on real-world enterprise text-to-sql workflows. arXiv preprint arXiv:2411.07763 (2024).

[7] Gao, D. et al. Text-to-sql empowered by large language models: A benchmark evaluation. arXiv preprint arXiv:2308.15363 (2023).

[8] Katsogiannis-Meimarakis, G., Xydas, M. & Koutrika, G. Natural language interfaces for databases with deep learning. *Proc. VLDB Endowm.* **16**, 3878–3881 (2023).

[9] Zelle, J. M. & Mooney, R. J. Learning to parse database queries using inductive logic programming. In *Proceedings of the national conference on artificial intelligence*, 1050–1055 (1996).

[10] Saha, D. et al. Athena: an ontology-driven system for natural language querying over relational data stores. *Proc. VLDB Endow.* **9**, 1209–1220 (2016).

[11] Guo, J. et al. Towards complex text-to-sql in cross-domain database with intermediate representation. arXiv preprint arXiv:1905.08205 (2019).

[12] Choi, D., Shin, M. C., Kim, E. & Shin, D. R. Ryansql: Recursively applying sketch-based slot fillings for complex text-to-sql in cross-domain databases. *Computat. Lingu.* **47**, 309–332 (2021).

[13] Wang, B., Shin, R., Liu, X., Polozov, O. & Richardson, M. Rat-sql: Relation-aware schema encoding and linking for text-to-sql parsers. arXiv preprint arXiv:1911.04942 (2019).

[14] Cao, R. et al. Lgesql: line graph enhanced text-to-sql model with mixed local and non-local relations. arXiv preprint arXiv:2106.01093 (2021).

[15] Raffel, C. et al. Exploring the limits of transfer learning with a unified text-to-text transformer. *J. Mach. Learn. Res.* **21**, 1–67 (2020).34305477

[16] Scholak, T., Schucher, N. & Bahdanau, D. Picard: Parsing incrementally for constrained auto-regressive decoding from language models. arXiv preprint arXiv:2109.05093 (2021).

[17] Li, H., Zhang, J., Li, C. & Chen, H. Resdsql: decoupling schema linking and skeleton parsing for text-to-sql. In *Proceedings of the Thirty-Seventh AAAI conference on artificial intelligence and thirty-fifth conference on innovative applications of artificial intelligence and thirteenth symposium on educational advances in artificial intelligence*, AAAI’23/IAAI’23/EAAI’23, 10.1609/aaai.v37i11.26535 (AAAI Press, 2023).

[18] Li, H. et al. Codes: Towards building open-source language models for text-to-sql. *Proc. ACM Manag. Data* **2**, 1–28 (2024).

[19] Brown, T. B. Language models are few-shot learners. arXiv preprint arXiv:2005.14165 (2020).

[20] Achiam, J. et al. Gpt-4 technical report. arXiv preprint arXiv:2303.08774 (2023).

[21] Dubey, A. et al. The llama 3 herd of models. arXiv preprint arXiv:2407.21783 (2024).

[22] Team, G. et al. Gemma 2: Improving open language models at a practical size. arXiv preprint arXiv:2408.00118 (2024).

[23] Abdin, M. et al. Phi-3 technical report: A highly capable language model locally on your phone. arXiv preprint arXiv:2404.14219 (2024).

[24] Jiang, A. Q. et al. Mistral 7b. arXiv preprint arXiv:2310.06825 (2023).

[25] Jiang, A. Q. et al. Mixtral of experts. arXiv preprint arXiv:2401.04088 (2024).

[26] Min, S. et al. Rethinking the role of demonstrations: What makes in-context learning work? arXiv preprint arXiv:2202.12837 (2022).

[27] Liu, A., Hu, X., Wen, L. & Yu, P. S. A comprehensive evaluation of chatgpt’s zero-shot text-to-sql capability. arXiv preprint arXiv:2303.13547 (2023).

[28] Rajkumar, N., Li, R. & Bahdanau, D. Evaluating the text-to-sql capabilities of large language models. arXiv preprint arXiv:2204.00498 (2022).

[29] Wei, J. et al. Chain-of-thought prompting elicits reasoning in large language models. *Adv. Neural Inf. Process. Syst.* **35**, 24824–24837 (2022).

[30] Liu, J. et al. What makes good in-context examples for gpt-? arXiv preprint arXiv:2101.06804 (2021).

[31] Dong, X. et al. C3: Zero-shot text-to-sql with chatgpt. arXiv preprint arXiv:2307.07306 (2023).

[32] Dahl, D. A. et al. Expanding the scope of the atis task: The atis-3 corpus. In *Human language technology: Proceedings of a workshop held at Plainsboro, New Jersey, March 8-11, 1994* (1994).

[33] Iyer, S., Konstas, I., Cheung, A., Krishnamurthy, J. & Zettlemoyer, L. Learning a neural semantic parser from user feedback. arXiv preprint arXiv:1704.08760 (2017).

[34] Zhong, V., Xiong, C. & Socher, R. Seq2sql: Generating structured queries from natural language using reinforcement learning. arXiv preprint arXiv:1709.00103 (2017).

[35] Yu, T. et al. Spider: A large-scale human-labeled dataset for complex and cross-domain semantic parsing and text-to-sql task. arXiv preprint arXiv:1809.08887 (2018).

[36] Hazoom, M., Malik, V. & Bogin, B. Text-to-sql in the wild: A naturally-occurring dataset based on stack exchange data. arXiv preprint arXiv:2106.05006 (2021).

[37] Wang, P., Shi, T. & Reddy, C. K. Text-to-sql generation for question answering on electronic medical records. *Proc. Web Conf.* **2020**, 350–361 (2020).

[38] Lee, G. et al. Ehrsql: A practical text-to-sql benchmark for electronic health records. *Adv. Neural Inf. Process. Syst.* **35**, 15589–15601 (2022).

[39] Lee, C.-H., Polozov, O. & Richardson, M. Kaggledbqa: Realistic evaluation of text-to-sql parsers. arXiv preprint arXiv:2106.11455 (2021).

[40] Gan, Y., Chen, X. & Purver, M. Exploring underexplored limitations of cross-domain text-to-sql generalization. arXiv preprint arXiv:2109.05157 (2021).

[41] Lan, W. et al. Unite: A unified benchmark for text-to-sql evaluation. arXiv preprint arXiv:2305.16265 (2023).

[42] Zhang, Y. et al. Sciencebenchmark: A complex real-world benchmark for evaluating natural language to sql systems. arXiv preprint arXiv:2306.04743 (2023).

[43] Zhang, T., Kishore, V., Wu, F., Weinberger, K. Q. & Artzi, Y. Bertscore: Evaluating text generation with bert. arXiv preprint arXiv:1904.09675 (2019).

[44] Zhang, B. et al. Benchmarking the text-to-sql capability of large language models: A comprehensive evaluation. arXiv preprint arXiv:2403.02951 (2024).

[45] Li, B., Luo, Y., Chai, C., Li, G. & Tang, N. The dawn of natural language to sql: Are we fully ready? arXiv preprint arXiv:2406.01265 (2024).

[46] Li, X. & Li, J. Angle-optimized text embeddings. arXiv preprint arXiv:2309.12871 (2023).

[47] Kendall, M. G. A new measure of rank correlation. *Biometrika* **30**, 81–93 (1938).

