# Supplementary material for: Redefining text-to-SQL metrics by incorporating semantic and structural similarity
Source: Sci Rep. 2025 Jul 1;15:22357. doi: 10.1038/s41598-025-04890-9 (PMC12216993; doi:10.1038/s41598-025-04890-9)
Supplement: Supplementary file 1 — Supplementary Information. [file 41598_2025_4890_MOESM1_ESM.pdf]

Supplementary material: Redefining Text-to-SQL Metrics by Incorporating Semantic and Structural Similarity

Supplementary material

In this supplementary material, we present all the SQL queries used to evaluate the proposed metric. These queries belong to groups Column Number, Row Number, Columns with Additional Rows, Rows with Additional Columns, Rows and Columns Combined and Rows with Different Ordering, as introduced in Section 5.5. In group Column Number, we incremented the number of columns and left the number of rows unchanged.

```
SELECT mailstreet
FROM schools
WHERE cdscode IN (
    SELECT cdscode
    FROM frpm
    ORDER BY "frpm count (k-12)" DESC
    LIMIT 1
    );
```

Query 1. Group 1 - query 1: one column, one row, and descending order.

```
SELECT mailstreet, mailstrabr
FROM schools
WHERE cdscode IN (
    SELECT cdscode
    FROM frpm
    ORDER BY "frpm count (k-12)" DESC
    LIMIT 1
    );
```

Query 2. Group 1 - query 2: two columns, one row, and descending order.

```
SELECT mailstreet, mailstrabr, mailcity
FROM schools
WHERE cdscode IN (
    SELECT cdscode
    FROM frpm
    ORDER BY "frpm count (k-12)" DESC
    LIMIT 1
    );
```

Query 3. Group 1 - query 3: three columns, one row, and descending order.

```
SELECT mailstreet, mailstrabr, mailcity, mailzip
FROM schools
WHERE cdscode IN (
    SELECT cdscode
    FROM frpm
    ORDER BY "frpm count (k-12)" DESC
    LIMIT 1
    );
```

Query 4. Group 1 - query 4: four columns, one row, and descending order.

In group Row Number, we incremented the number of rows and leaved the number of columns unchanged.

```
SELECT mailstreet
FROM schools
WHERE cdscode IN (
    SELECT cdscode
    FROM frpm
    ORDER BY "frpm count (k-12)" DESC
    LIMIT 1
    );
```

Query 5. Group 2 - query 1: one column, one row, and descending order.

```
SELECT mailstreet
FROM schools
WHERE cdscode IN (
    SELECT cdscode
    FROM frpm
    ORDER BY "frpm count (k-12)" DESC
    LIMIT 2
    );
```

Query 6. Group 2 - query 2: one column, two rows, and descending order.

```
SELECT mailstreet
FROM schools
WHERE cdscode IN (
    SELECT cdscode
    FROM frpm
    ORDER BY "frpm count (k-12)" DESC
    LIMIT 3
    );
```

Query 7. Group 2 - query 3: one column, three rows, and descending order.

```
SELECT mailstreet
FROM schools
WHERE cdscode IN (
    SELECT cdscode
    FROM frpm
    ORDER BY "frpm count (k-12)" DESC
    LIMIT 4
    );
```

Query 8. Group 2 - query 4: one column, four rows, and descending order.

In group Columns with Additional Rows, we examines how the metric behaves as the number of columns increases from 1 to 4, while maintaining a constant number of rows at 4.

```
SELECT mailstreet
FROM schools
WHERE cdscode IN (
    SELECT cdscode
    FROM frpm
    ORDER BY "frpm count (k-12)" DESC
    LIMIT 4
    );
```

Query 9. Group 3 - query 1: one column, four rows, and descending order.

```
SELECT mailstreet, mailstrabr
FROM schools
WHERE cdscode IN (
    SELECT cdscode
    FROM frpm
    ORDER BY "frpm count (k-12)" DESC
    LIMIT 4
    );
```

Query 10. Group 3 - query 2: two columns, four rows, and descending order.

```
SELECT mailstreet, mailstrabr, mailcity
FROM schools
WHERE cdscode IN (
    SELECT cdscode
    FROM frpm
    ORDER BY "frpm count (k-12)" DESC
    LIMIT 4
    );
```

Query 11. Group 3 - query 3: three columns, four rows, and descending order.

```
SELECT mailstreet, mailstrabr, mailcity, mailzip
FROM schools
WHERE cdscode IN (
    SELECT cdscode
    FROM frpm
    ORDER BY "frpm count (k-12)" DESC
    LIMIT 4
    );
```

Query 12. Group 3 - query 4: four columns, four rows, and descending order.

In group Rows with Additional Columns, we examines how the metric behaves as the number of rows increases from 1 to 4, while maintaining a constant number of columns at 4.

```
SELECT mailstreet, mailstrabr, mailcity, mailzip
FROM schools
WHERE cdscode IN (
    SELECT cdscode
    FROM frpm
    ORDER BY "frpm count (k-12)" DESC
    LIMIT 1
    );
```

Query 13. Group 4 - query 1: one column, four rows, and descending order.

```
SELECT mailstreet, mailstrabr, mailcity, mailzip
FROM schools
WHERE cdscode IN (
    SELECT cdscode
    FROM frpm
    ORDER BY "frpm count (k-12)" DESC
    LIMIT 2
    );
```

Query 14. Group 4 - query 2: two columns, four rows, and descending order.

```
SELECT mailstreet, mailstrabr, mailcity, mailzip
FROM schools
```

```sql
WHERE cdscode IN (
    SELECT cdscode
    FROM frpm
    ORDER BY "frpm count (k-12)" DESC
    LIMIT 3
    );
```

Query 15. Group 4 - query 3: three columns, four rows, and descending order.

```sql
SELECT mailstreet, mailstrabr, mailcity, mailzip
FROM schools
WHERE cdscode IN (
    SELECT cdscode
    FROM frpm
    ORDER BY "frpm count (k-12)" DESC
    LIMIT 4
    );
```

Query 16. Group 4 - query 4: four columns, four rows, and descending order.

In group Rows and Columns Combined, we simultaneously increased both rows and columns.

```sql
SELECT mailstreet
FROM schools
WHERE cdscode IN (
    SELECT cdscode
    FROM frpm
    ORDER BY "frpm count (k-12)" DESC
    LIMIT 1
    );
```

Query 17. Group 5 - query 1: one column, one row, and descending order.

```sql
SELECT mailstreet, mailstrabr
FROM schools
WHERE cdscode IN (
    SELECT cdscode
    FROM frpm
    ORDER BY "frpm count (k-12)" DESC
    LIMIT 2
    );
```

Query 18. Group 5 - query 2: two columns, two rows, and descending order.

```sql
SELECT mailstreet, mailstrabr, mailcity
FROM schools
WHERE cdscode IN (
    SELECT cdscode
    FROM frpm
    ORDER BY "frpm count (k-12)" DESC
    LIMIT 3
    );
```

Query 19. Group 5 - query 3: three columns, three rows, and descending order.

```sql
SELECT mailstreet, mailstrabr, mailcity, mailzip
FROM schools
WHERE cdscode IN (
    SELECT cdscode
    FROM frpm
    ORDER BY "frpm count (k-12)" DESC
```

```
    LIMIT 4
    );
```

Query 20. Group 5 - query 4: four columns, four rows, and descending order.

In group Rows with Different Ordering, we switched the order from descending (DESC) to ascending (ASC) and progressively increased the number of rows.

```
SELECT mailstreet
FROM schools
WHERE cdscode IN (
    SELECT cdscode
    FROM frpm
    ORDER BY "frpm count (k-12)" ASC
    LIMIT 1
    );
```

Query 21. Group 6 - query 1: one column, one row, and ascending order.

```
SELECT mailstreet
FROM schools
WHERE cdscode IN (
    SELECT cdscode
    FROM frpm
    ORDER BY "frpm count (k-12)" ASC
    LIMIT 2
    );
```

Query 22. Group 6 - query 2: one column, two rows, and ascending order.

```
SELECT mailstreet
FROM schools
WHERE cdscode IN (
    SELECT cdscode
    FROM frpm
    ORDER BY "frpm count (k-12)" ASC
    LIMIT 3
    );
```

Query 23. Group 6 - query 3: one column, three rows, and ascending order.

```
SELECT mailstreet
FROM schools
WHERE cdscode IN (
    SELECT cdscode
    FROM frpm
    ORDER BY "frpm count (k-12)" ASC
    LIMIT 4
    );
```

Query 24. Group 6 - query 4: one column, four rows, and ascending order.
